# Supplementary material for: Surface Microbiome Profiling of Dental Elevators Using Third‐Generation Sequencing: Implications for Infection Control in Dental Practice
Source: Microbiologyopen. 2025 Dec 8;14(6):e70178. doi: 10.1002/mbo3.70178 (PMC12685768; doi:10.1002/mbo3.70178)
Supplement: Supplementary file 1 — customer_backup. [file MBO3-14-e70178-s001.zip › customer_backup/customer_backup/function_analysis/COG/allsample/K2species/allsample.reabundance_Class2Order.txt.pdf]

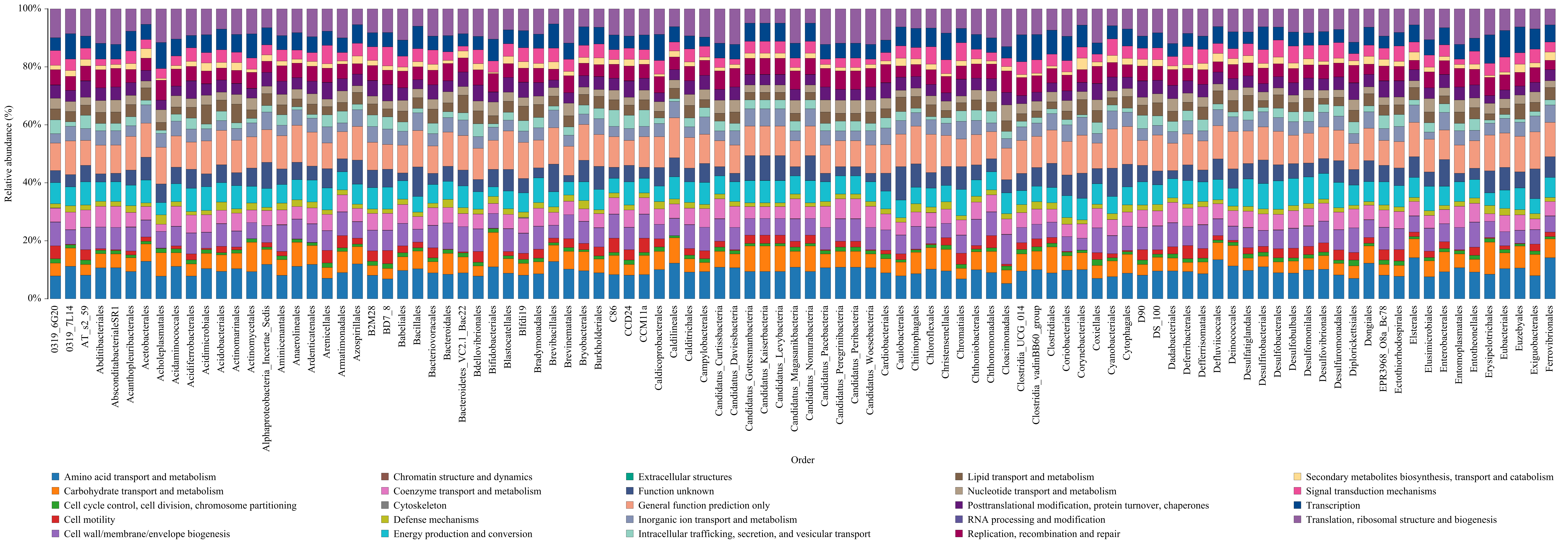
Relative abundance (%)
100%
80%
60%
40%
20%
0%
0319_6G20
0319_7L14
AT_s2_59
Abditibacteriales
AbsconditabacterialesSR1
Acanthopleuribacterales
Acetobacterales
Acholeplasmatales
Acidaminococcales
Acidiferrobacterales
Acidimicrobiales
Acidobacteriales
Actinomarinales
Actinomycetales
Alphaproteobacteria_Incertae_Sedis
Aminicenantales
Anaerolineales
Ardenticatenales
Arenicellales
Armatimonadales
Azospirillales
B2M28
BD7_8
Babeliales
Bacillales
Bacteriovoracales
Bacteroidales
Bacteroidetes_VC2.1_Bac22
Bdellovibrionales
Bifidobacteriales
Blastocatellales
Bifdi19
Bradymonadales
Brevibacillales
Brevinematales
Bryobacterales
Burkholderiales
C86
CCD24
CCM11a
Caldicoprobacterales
Caldilineales
Calditrichales
Campylobacterales
Candidatus_Curtissbacteria
Candidatus_Daviesbacteria
Candidatus_Gottesmanbacteria
Candidatus_Kaiserbacteria
Candidatus_Levybacteria
Candidatus_Magasanikbacteria
Candidatus_Nomurabacteria
Candidatus_Pacebacteria
Candidatus_Peregrinibacteria
Candidatus_Peribacteria
Candidatus_Woesebacteria
Cardiobacteriales
Caulobacterales
Chitinophagales
Chloroflexales
Christensenellales
Chromatiales
Chthoniobacterales
Chthonomonadales
Cloacimonadales
Clostridia_UCG_014
Clostridia_vadinBB60_group
Clostridiales
Coriobacteriales
Corynebacteriales
Coxiellales
Cyanobacteriales
Cytophagales
D90
DS_100
Dadabacteriales
Deferribacterales
Defferrisomatales
Defluviicoccales
Deinococcales
Desulfatiglandales
Desulfitobacteriales
Desulfobacterales
Desulfobulbales
Desulfomonilales
Desulfovibrionales
Desulfuromonadales
Diplorickettsiales
Dongiales
EPR3968_O8a_Bc78
Ectothiorhodospirales
Elsterales
Elusimicrobiales
Enterobacterales
Entomoplasmatales
Entotheonellales
Erysipelotrichales
Eubacteriales
Euzebyales
Exiguobacterales
Ferrovibrionales
Order
Amino acid transport and metabolism
Carbohydrate transport and metabolism
Cell cycle control, cell division, chromosome partitioning
Cell motility
Cell wall/membrane/envelope biogenesis
Chromatin structure and dynamics
Coenzyme transport and metabolism
Cytoskeleton
Defense mechanisms
Energy production and conversion
Extracellular structures
Function unknown
General function prediction only
Inorganic ion transport and metabolism
Intracellular trafficking, secretion, and vesicular transport
Lipid transport and metabolism
Nucleotide transport and metabolism
Posttranslational modification, protein turnover, chaperones
RNA processing and modification
Replication, recombination and repair
Secondary metabolites biosynthesis, transport and catabolism
Signal transduction mechanisms
Transcription
Translation, ribosomal structure and biogenesis
